# Efficacy and Safety of Radiofrequency Ablation for Breast Cancer Smaller Than 2 cm: A Systematic Review and Meta-Analysis

**DOI:** 10.3389/fonc.2021.651646

**Published:** 2021-05-03

**Authors:** Lin-Yu Xia, Qing-Lin Hu, Wei-Yun Xu

**Affiliations:** ^1^ Department of Thyroid and Breast Surgery, The First Affiliated Hospital of Chengdu Medical College, Chengdu, China; ^2^ Department of Breast Surgery, Mianyang Central Hospital, Mianyang, China

**Keywords:** breast cancer, radiofrequency ablation, effective, safe, meta-analysis

## Abstract

**Background:**

To evaluate the efficacy and safety of radiofrequency ablation (RFA) of breast cancer smaller than 2 cm.

**Methods:**

A systematic search was conducted in the PubMed and EMBASE databases to identify published studies investigating the efficacy and safety of RFA for breast cancer smaller than 2 cm. The main outcomes were technical success rate of the ablation, complete ablation rate, complications and local recurrence. Secondary considerations were mode of anesthesia, pain tolerance, mean ablation time and surgical excision after ablation.

**Results:**

Seventeen studies involving 399 patients and 401 lesions met the inclusion criteria. Nearly 99%(95%CI=0.98-1.00) of lesions achieved good technical success rate.Notably, 83.88% of the patients received RFA under general anesthesia (333/397) whereas 15.87% received RFA under local anesthesia (63/397). Of the 63, 98.41% tolerated the pain associated with the procedure. Majority of patients (65.74%, 261/397) underwent surgical excision of the tumor after ablation whereas in a few patients (34.26%, 136/397), the tumor tissue was retained in the breast after ablation. Complete ablation was achieved in 96% of patients for a mean time of 15.8 minutes (95%CI=0.93-0.99). Overall, only 2% (95%CI=0.01-0.04) of the individuals developed complications. Skin burns (2.02%, 8/397) were the most common complications. There was no local recurrence after a median follow-up of 27.29 months, whether or not they underwent surgical resection following RFA.

**Conclusion:**

The results show that RFA for breast cancer smaller than 2 cm is safe and effective. However, prospective studies are needed to validate this conclusion.

## Introduction

Globally, breast cancer is the most prevalent malignant cancer among women (1). The need for quality life and better aesthetics has reduced the application of invasive cancer interventions. In recent years, RFA has received increasing attention because of its noninvasive nature in the treatment of breast cancer. RFA utilizes radiofrequency alternating current to ablate tissue around a needle electrode, resulting in local coagulative tumor necrosis (2). Previous studies have demonstrated that RFA is a safe and effective (63% to 96%) local treatment of breast cancer, but these findings are based on tumors with size ranging from 0.2cm to 5cm (3–5). Three meta-analyses provided preliminary evidence that RFA can effectively treat breast cancer (6–8). Of the three studies, only one was specific for RFA, and the other two combined five ablation technologies, including microwave, laser, radiofrequency, high-intensity focused ultrasound and cryoablation. Although surgical resection performed after ablation affects the complete ablation rate, occurrence of complications and local recurrence, but they did not perform subgroup analyses to determine this. Most importantly, none of the three meta-analyses limited the size of tumors, an important factor affecting safety and effectiveness of breast tumor ablation. Many studies have shown that RFA is most effective in treating small tumors (9–11). To capture evidence from recent research and eliminate the impact of heterogeneity of tumor size on the results, this meta-analysis was performed focusing on breast tumors smaller than 2 cm.

## Methods

### Search Strategy

A systematic literature search was performed on the PubMed and Embase databases to identify studies published up to October 1, 2020. The following key terms were used for the search: (“breast cancer, OR breast neoplasm” AND “radiofrequency ablation” OR “radiofrequency” OR “ablation”). The search was not limited to any geographical region or language.

### Inclusion and Exclusion Criteria

The inclusion criteria were; 1) patients diagnosed with breast cancer; 2) patients who underwent RFA, regardless of whether there was surgical resection after ablation, immediate resection or delayed resection; 3) studies including at least one clinical outcome (rate of successful ablation, rate of complete ablation, incidences of complications or recurrence). Exclusion criteria were; 1) review articles, letters to the editor, comments, editorials and case reports; 2) Lack of clinical outcome data; 3) studies in which some tumors were larger than 2 cm.

### Study Selection and Data Extraction

Data extraction was performed by researchers working independently using a standardized data extraction form. Eligible studies were carefully and systematically reviewed. The following information was obtained: author’s name, year of publication, nationality of patients, number and age of participants, the size of tumor, tumor receptor, histology, nottingham grade, lymph node metastasis, means of image guidance, mode of anesthesia, pain tolerance, mean ablation time, surgical excision, pathological evaluation, median follow-up time, technically successful ablation rate, rate of complete ablation, complications and local recurrence. Technical success rate of ablation was defined as the completion of ablation treatment in terms of technology and good cooperation from patients. Complete ablation was defined as ablation of all tumor tissues as evidenced from imaging and pathological examination, or complete necrosis of tumor tissues after ablation. Local recurrence was defined as incomplete local treatment and recurrence in the breast distant from the ablation zone.

### Quality Assessment

Eight items from the non-randomized experimental research-MINORS scale established by Slim et al. were used to evaluate the quality of the selected literature (12). Study quality was scored as 0 (not reported), 1 (inadequately reported), or 2 (adequately reported). A score greater than 12 was considered high quality, 8-12 was considered medium quality, and less than 8 was considered low quality. To ensure quality results, we only retained studies with scores of 8 or more (**Supplementary Table 1**).

### Summary Measures and Statistical Analysis

The principal outcomes were technical success rate, complete ablation rate and rate of complications. These rates were combined to perform subgroup analysis based on whether surgical resection was performed after ablation. Meta-analysis was conducted using R Project (R version 3.6.2 for Windows) with the “meta” package. Chi-squared and I^2^ were used to measure statistical heterogeneity among the studies. When I^2^>50%, the studies were considered to have significantly high heterogeneity, thus, random-effects model was used (13). Otherwise, the fixed-effects model was used. All tests were two-sided with a *P *< 0.05 considered statistically significant. Publication bias was evaluated using a funnel plot and Egger’s test (14). Trim and fill analyses were performed if there was publication bias.

## Results

### Study Selection and the Characteristics of Studies

**Figure 1:**
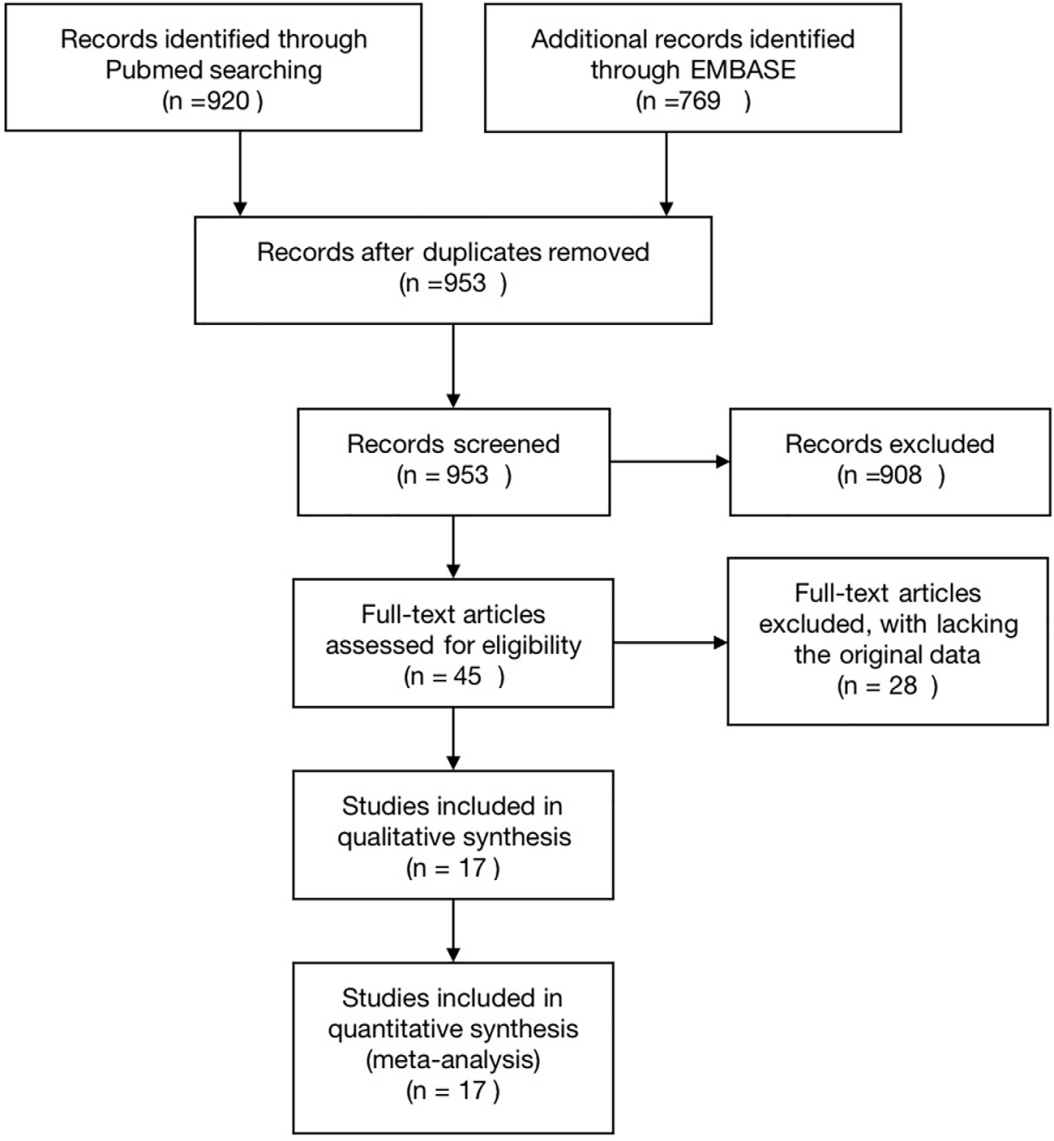
Flowchart explaining the article selection.

A total of 1689 articles were identified, among which 953 were selected after removal of duplicates. After reading the title or abstract, 908 articles irrelevant to this study were also removed. Based on the inclusion and exclusion criteria, 28 more articles were excluded. In the end, 17 studies covering 399 patients and 401 lesions were included in the final analyses (10, 11, 15–29). All included studies were experimental studies. The study selection process for the meta-analysis is shown in **Figure 1**.

Characteristics of the included studies are shown in **Table 1**. The number of patients in individual studies ranged from 3 to 52, and aged between 33 to 92 years old. The size of the tumors ranged between 0.4 to 2 cm. Collectively, 399 patients and 401 lesions were analyzed as shown in **Table 2**.

**Table 1:** Patient and tumor characteristics in the trials included in the meta-analysis.

Authors	Year	Country	N(patients/lesions)	Mean age(range, years)	Tumor size(cm)	ER(+/-),PR(+/-),HER2(+/-)	Histology(IDC/ILC/others)	Axillary status(N0/N1)	NG(1/2/3)	AST	RT
Burak (10)	2003	USA	10/10	53.70 (37–67)	1.20 (0.80–1.60)	6/4,6/4,0/10	NR	NR	2003/3/4	NR	NR
Fornage (11)	2004	USA	20/21	56 (38–80)	<2	NR	19/2/0	16/4	NR	NR	NR
Noguch (15)	2006	Japan	10/10	54 (33–70)	1.10 (0.50–2)	8/2,5/5,1/9	7/0/3	10/0	NR	NR	NR
Susini (16)	2007	Italy	3/3	81.30(76-86)	1.16 (1–1.30)	NR	3/0/0	NR	0/1/2	0	0
Khatri (17)	2007	USA	17/17	63 (39–83)	1.30 (0.80–1.50)	14/1,13/2,NR	14/1/0	12/2	NR	NA	13
Oura (18)	2007	Japan	52/52	55 (37–83)	1.30 (0.50–2)	NR	7/42/3	43/9	NR	52	52
Manenti (19)	2009	Italy	34/34	53 (49–62)	<2	NR	NR	27/7	NR	34	34
Nagashima (20)	2009	Japan	17/17	62(47–71)	1.10 (0.60–1.80)	12/5,14/3,NR	NA	14/3	9/6/2	17	17
Wiksell (21)	2010	Sweden	33/33	NR (46–83)	0.60–1.50	NR	26/2/5	NR	8/23/1	NR	NR
Yamamoto (22)	2011	Japan	29/30	55.90 (38–78)	1.28 (0.50–1.90)	NR	NR	NR	NR	NA	30
Ohtani (23)	2011	Japan	41/41	59 (38–92)	<2	38/3,35/6,1/40	36/0/5	41/0	26/3/2	41	41
Yoshinaga (24)	2012	Japan	14/14	NR (45–82)	0.60–2	6/2,5/3,0/8*	8/0/0*	7/1*	6/2/0*	7*	8*
Manenti (25)	2013	Italy	40/40	<2	<2	NA	40/0/0	NR	NA	40	40
Waaijer (26)	2014	Sweden	15/15	NR (50–76)	1.10(0.40-1.70)	12/3,10/5,0/15	10/0/5	NR	6/6/3	7	9
Schässburger (27)	2014	Sweden	18/18	NR (46–84)	0.50-2	18/0,16/2,0/18	NA	17/1	5/13/0	18	17
Nagashima (28)	2015	Japan	26/26	NR	<2	NA	NA	NA	NA	NR	NR
García (29)	2018	Spain	20/20	64(46–86)	<2	NA	NA	14/6	10/7/3	19	6

IDC, invasive ductal carcinoma; ILC, invasive lobular carcinoma; NG, nottingham grade; AST, adjuvant systemic therapy; RT, radiation therapy; NR, not reported; NA, not applicable; *The data collected were only from 8 patients who did not receive surgical resection.

**Table 2:** The treatment characteristics and complications of radiofrequency ablation in the trials.

Authors	IG	Electrode probe	AM	Pain tolerance	Mean time RFA(min)	Surgical excision	Pathologic evaluation	Follow-up	Complications
Burak (10)	US	Le Veen needle	L	well	13.8	delayed	H&E	NR	0
Fornage (11)	US	Starburst XL	G	NA	21.2	Immediate	H&E,NADH	NR	0
Noguch (15)	US	Starburst XL	G	NA	18	Immediate	H&E,NADH	NR	0
Susini (16)	US	Cool-Tip	L	well	10.3	not	NR	9mon	0
Khatri (17)	US	Cool-Tip	G	NA	21	Immediate	NADH	25mon	2 pt skin puckering,1 pt breast infections
Oura (18)	US	Cool-Tip	G	NA	12	not	NR	15mon	1 pt skin burn
Manenti (19)	US	Cool-Tip	G	NA	27 ± 3.7	delayed	H&E,NADH	NR	1 pt skin burn
Nagashima (20)	US	Cool-Tip	G	NA	9.6	not	H&E	19mon	0
Wiksell (21)	US	NR	G	NA	9.5 ± 1.2	Immediate	H&E	NR	1 pt skin burn, 2 pt skin swelling,1 pt chest muscle burn,1 pt pneumothorax
Yamamoto (22)	US	Cool-Tip	G	NA	11.4	not	H&E,NADH	17mon	3 pt skin burn,1 pt breast lesion
Ohtani (23)	US	Cool-Tip	32 pt L, 9 pt G	1 pt intolerable	9	delayed	H&E,NADH	NR	1 pt skin burn
Yoshinaga (24)	US	Cool-Tip	G	NA	9.6	6 pt immediate, 8 pt not	NADH	39.9mon	1 pt skin burn
Manenti (25)	US	Cool-Tip	G	NA	27 ± 3.7	delayed	H&E,NADH	18mon	0
Waaijer (26)	US	NR	NR	NA	13 ± 0.2	Immediate	H&E,CK8	17mon	1 pt pneumothorax
Schässburger (27)	US	internally cooled	L	well	10	delayed	H&E,CK8	NR	0
Nagashima (28)	US	NR	G	NA	NR	not	H&E	88mon	2 pt nipple retraction
García (29)	US	Cool-Tip	NR	NA	NR	Immediate	H&E,CK18	25mon	5 pt breast inflammation,3 pt breast infections

IG, image guidance; AM, anesthesia mode; TSAA, technically successful ablation rate; CAA, complete ablation rate; LR, local recurrence; L, local; G, general; pt, patient; NR, not report; NA, not applicable; mon, month; pt, patient.

### Technical Success Rate

**Figure 2:**
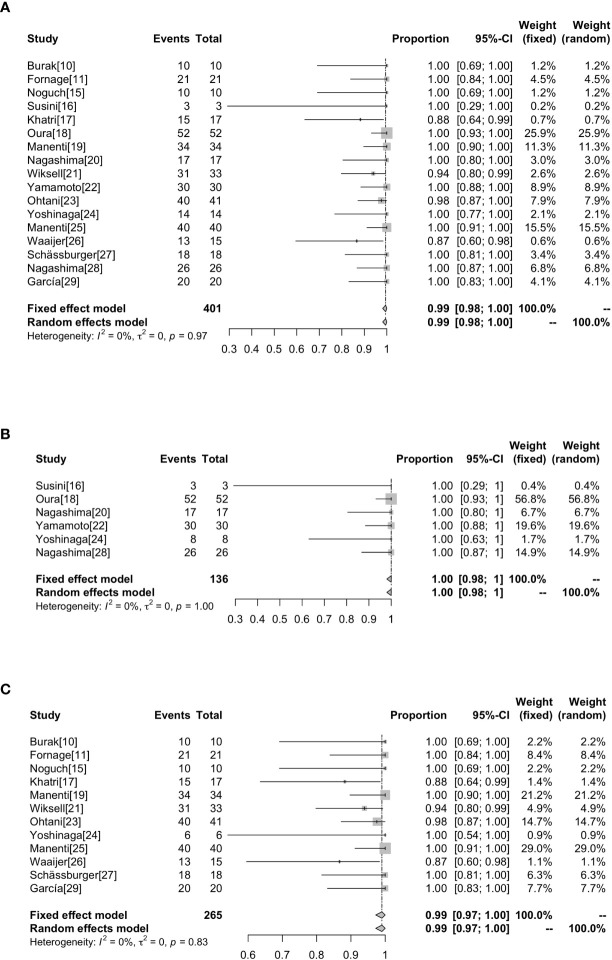
Forest plot showing the analysis of technically successful ablation rate in patients. The results of all the patients **(A)**, patients without surgical resection following RFA **(B)**, patients with surgical resection following RFA **(C)** are shown, respectively.

Technical success rate ranged from 86.67% to 100%. Of the 401 lesions, 7 (1.75%) were not successfully ablated (17, 21, 23, 26). Incomplete ablations were attributed to technical failure such as probe placement (0.75%,3/401) (21, 26), poor ultrasound imaging of the tumor (0.5%,2/401) (17, 21), uncooperative patients (0.25%,1/401) (17) and unbearable pain (0.25%,1/401) (23). Of the 7 patients who were not successfully ablated, 4 were excluded after pre-ablation assessment (17, 21), and 3 underwent ablation but did not complete the treatment (23, 26), so only 397 of the 401 lesions actually underwent ablation. Without heterogeneity (I^2^ = 0%, *P*=0.97), the combined technical success rate was 99% (95%CI=0.98-1.00). In the subgroup analysis based on whether surgical resection was performed after ablation, the success rate of ablation without surgical resection after ablation was 100% (95%CI=0.98-1.00, I^2^ = 0%, *P*=1), and the success rate of ablation with surgical operation following RFA was 99% (95%CI=0.97-1.00, I^2^ = 0%, *P*=0.83) as shown in **Figure 2**.

### The RFA and Surgical Excision

Ablation time ranged between 9 (23) to 30 minutes (19, 25) with an average of 15.8 minutes. Majority of patients, 333/397 (83.88%), received RFA under general anesthesia, whereas the rest, 63/397 (15.87%), received local anesthesia. Of the 63 patients who received local anesthesia, 62 reported tolerable pain whereas only 1 (1.59%) reported unbearable pain to the extent of not completing the ablation (23). These statistics are summarized in **Table 2**.

Majority of patients underwent surgical tumor excision after ablation (65.74%, 261/397). Of these, 118/261 (45.21%) received immediate excision whereas 143/261 (54.79%) received delayed excision. Of the 261, 241 (92.34%) underwent tumor resection (10, 11, 15, 17, 19, 21, 23–26, 29), whereas 20 (7.67%) underwent total mastectomy (10, 11, 15, 17, 27). Of the total patients analyzed in this study, 136 (34.26%) did not undergo surgical resection after tumor ablation (16, 18, 20, 22, 24, 28) (**Table 2**). Among the 136 patients who only received RFA, 133 patients received postoperative adjuvant radiotherapy and received systemic adjuvant therapy as needed according to the St. Gallen consensus except 3 patients who did not receive radiotherapy or systemic adjuvant therapy due to their poor age and general condition (16). Patients who received surgical resection after ablation also received radiotherapy or systemic adjuvant therapy as needed.

### Complete Ablation Rate

**Figure 3:**
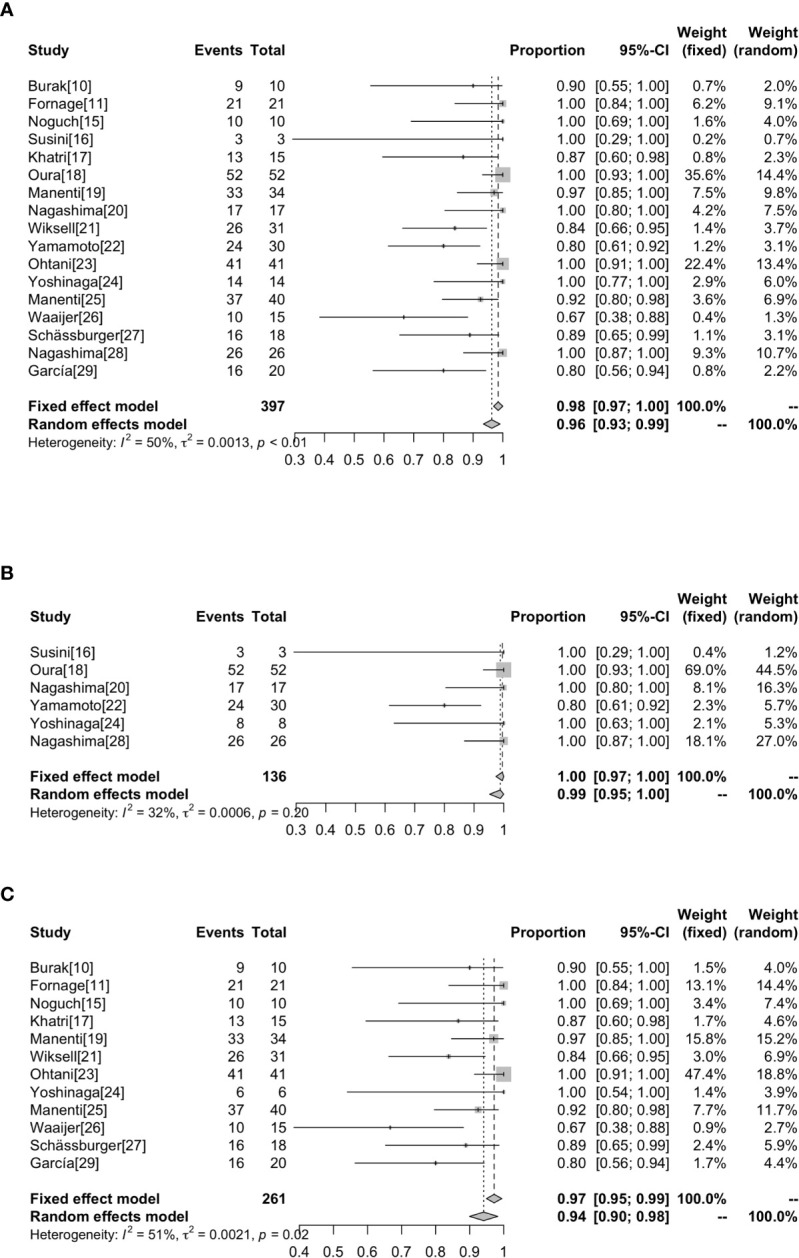
Forest plot showing the analysis of complete ablation rate in patients receiving RFA. The results of all the patients **(A)**, patients without surgical resection following RFA **(B)**, patients with surgical resection following RFA **(C)** are shown, respectively.

Whether the tumor is completely ablated is evaluated by imaging and pathological examination. At present, there is no uniform standard for the staining technology to evaluate the survival rate of tumor cells after RFA. The most widely used methods in our research are hemathoxylineosin (H & E) and NADH (nicotinamide adenine dinucleotide in its reduced form) diaphorase (**Table 2**). The highest complete ablation rate was 100% in 8 studies and the lowest complete ablation rate was 66.7% (26). Overall, the complete ablation rate was 98% (95%CI=0.97-1.00) without heterogeneity (I^2^ = 50%, *P*<0.01). Subgroup analysis showed that the rate of complete ablation without surgical resection after ablation was 100% (95%CI=0.97-1.00, I^2^ = 32%, *P*=0.20), whereas the rate of complete ablation with surgical resection after ablation was 94% (95%CI=0.90-0.98, I^2^ = 51%, *P*=0.02) (**Figure 3**).

### Complications

**Figure 4:**
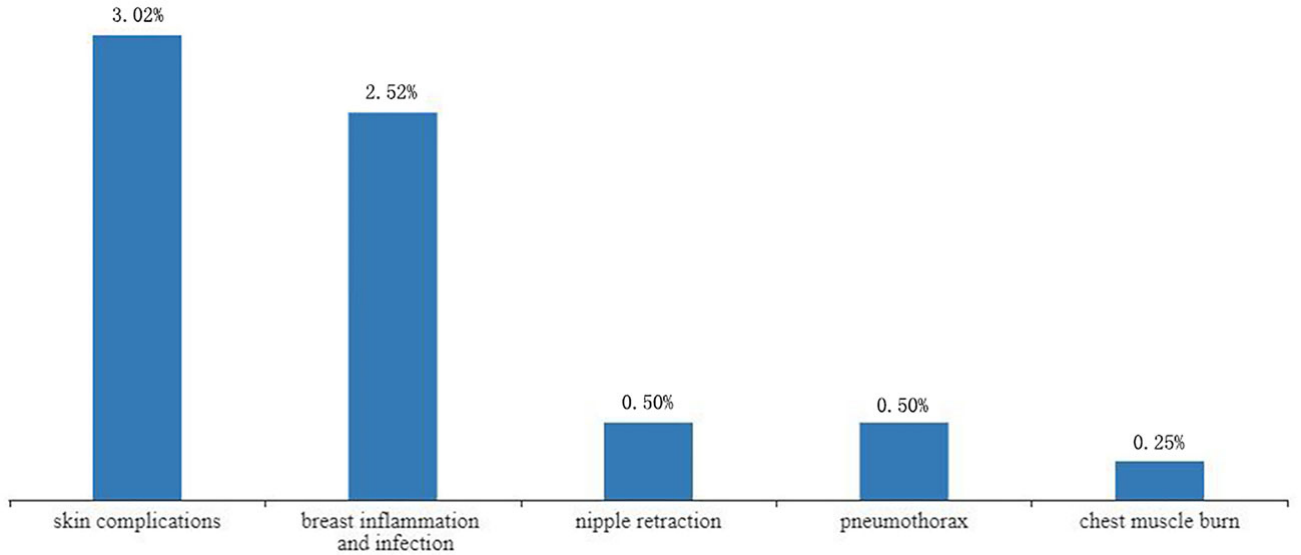
Complications rate in patients receiving RFA with different types.

**Figure 5:**
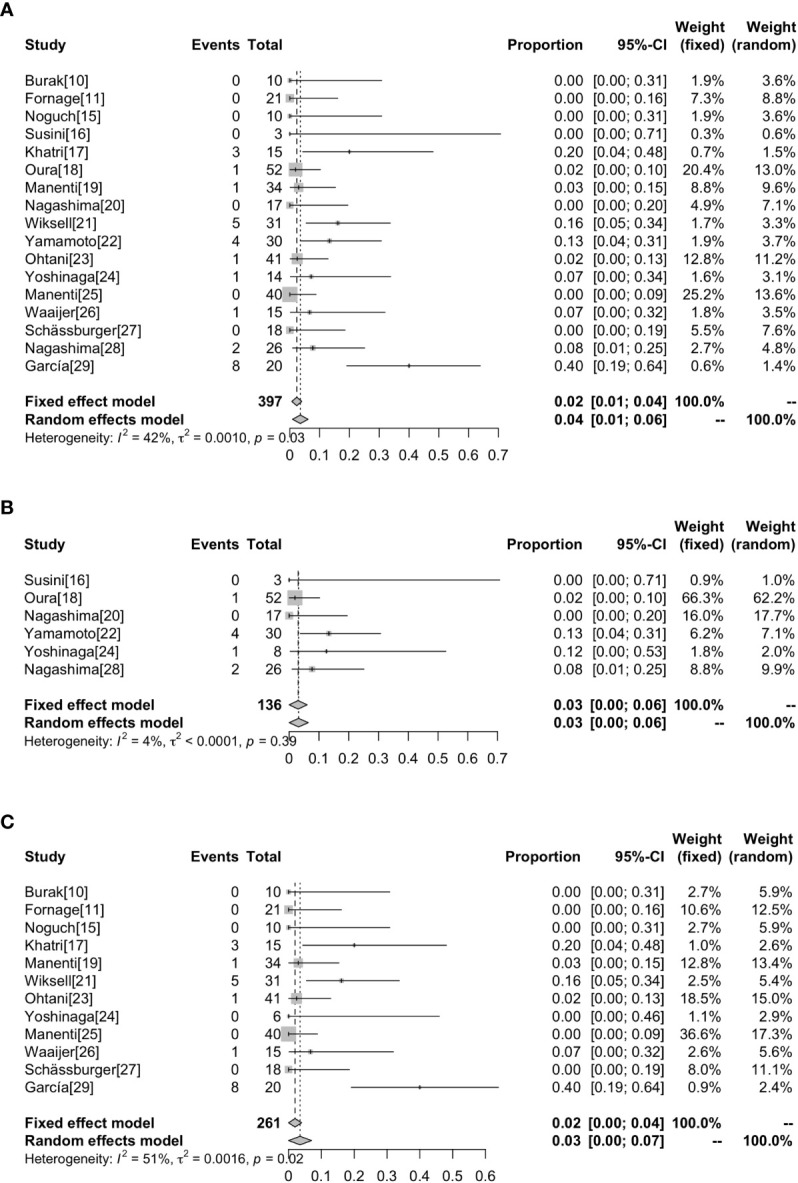
Forest plot showing the analysis of complications rate in patients receiving RFA. The results of all the patients **(A)**, patients without surgical resection following RFA **(B)**, patients with surgical resection following RFA **(C)** are shown, respectively.

Complications were reported in 17 studies. Of the 401 lesions included in the study, 397 received RFA, of which 27 developed complications (6.80%, 27/397) (17–19, 21–24, 26, 28, 29) (**Figure 4**). The most common complications was skin burn (2.02%, 8/397) (18, 19, 21–24), skin puckering (0.50%, 2/397) (17) and skin swelling (0.50%, 2/397) (21), some other complications are related to breast inflammation (1.51%, 6/397) (21, 29) and infections (1.01%, 4/397) (17, 29). Other complications included nipple retraction (0.50%, 2/397) (28), pneumothorax (0.50%, 2/397) (21, 26) and chest muscle burn (0.25%, 1/397) (21). Overall results revealed that the complications rate was 2% (95%CI=0.01-0.04) without heterogeneity (I^2^ = 42%, *P*=0.03). The results of subgroup analysis showed that the incidence of complications without surgical resection after ablation was 3% (95%CI=0.00-0.06, I^2^ = 4%, *P*=0.39), and the incidence of complications with surgical resection after ablation was 3% (95%CI=0.00-0.07, I^2^ = 51%, *P*=0.02) (**Figure 5**).

### Local Recurrence

Local recurrence after RFA was reported in 10 out of the 17 studies (16–18, 20, 22, 24–26, 28, 29). The median follow-up time ranged from 9 and 88 months. Local recurrence was evaluated according to clinical and imaging results. A total of 232 cases were reported whether they had local recurrence after RFA, including 136 cases who received RFA alone and 96 cases who underwent surgical resection after RFA. The median follow-up after ablation was 29 months for those who did not undergo surgical resection and 23 months for those who underwent surgical resection. There was no local recurrence after a median follow-up of 27.29 months in 232 patients, whether or not they underwent surgical resection following RFA.

**Figure 6:**
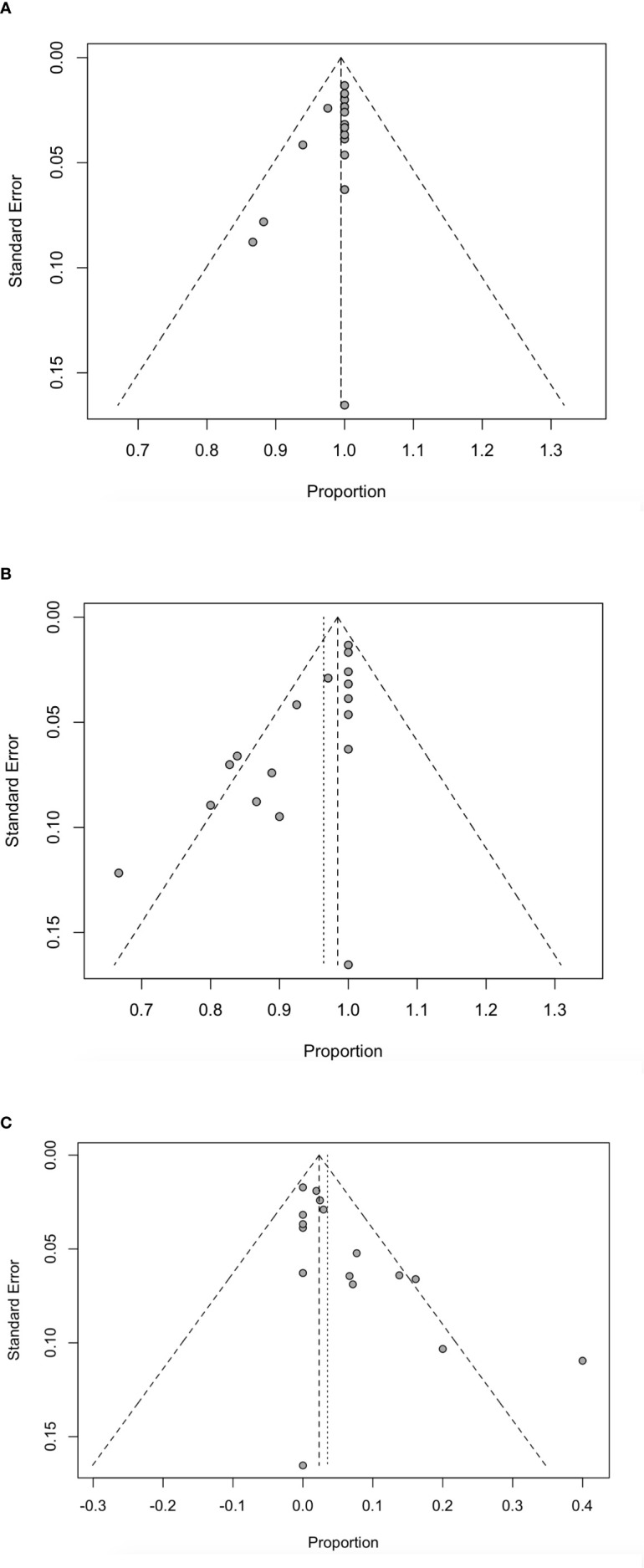
Funnel plot for technically successful ablation rate **(A)**, complete ablation rate **(B)** and complications rate **(C)** in patients receiving RFA.

Using funnel plots and Egger ‘s test, we analyzed publication bias on technical success rate, complete ablation rate and complication rate and the results are shown in **Figure 6**. All *P*-values were <0.05, indicating that there was potential publication bias (**Supplementary Table 2**). The impact of publication bias was assessed by trim and fill method. In the analysis of publication bias for the technical success rate, we found that there was no new study added, indicating that the conclusion was reliable and the effect of publication bias on the results was small. After addition of the estimated studies to the funnel plot of the complete ablation rate and complication rate, funnel plots become symmetrical, and the combined effect was still statistically significant (*P* <0.05) (**Supplementary Figure 1**).

## Discussion

RFA is a minimally invasive treatment technique for tumors. However, tumors larger than 3cm may not be successfully ablated by this procedure (30). In this research, 17 studies investigating RFA ablation for small breast tumors were analyzed. Overall, the technical success rate was 99% whereas the complete ablation rate was achieved in 96% of the cases. Complication rate was 2% of the cases. There was no local recurrence after a median follow-up of 27.29 months, whether or not the patients underwent surgical resection following RFA.

Accurate ultrasonic imaging and correct placement of the probe are the most important factors for a successful RFA (31). In RFA ablation, radiographic assistance is required to determine the location of the radiofrequency electrode needle inserted into the tumor as well as in monitoring the ablation effect in real time. In our analysis, 7 patients were not successfully ablated using RFA, partly attributed to poor tumor imaging and probe placement. Studies show that MRI is a promising guide for monitoring the electrode needle in RFA (32, 33). MRI can be used when a tumor cannot be clearly imaged by ultrasound. Our analysis excluded tumors larger than 2 cm, and found a higher technically successful ablation rate than that found by Mauri et al. (99% *vs.* 96%) (8).

Pathological examination of tumor tissue combined with imaging can assess the ablation status of a tumor. Since H & E cannot be used for immediate viability assessment after RFA (34, 35), evaluating NADH or cytokeratine 8 (CK8) has been preferred. H & E staining is most commonly used to assess the level of necrosis whereas NADH diaphorase or CK8 assays are used to assess tissue activity. In most patients (65.74%, 261/397), sample tissues were obtained by immediate or delayed local resection or total mastectomy after RFA. For those who retained the tumor tissues after ablation, samples were obtained by core needle biopsy (34.26%, 136/397). The complete ablation rate obtained in this study was higher than that reported by Chen et al. (96% *vs.* 89%) (7), indicating that the complete ablation rate of tumors less than 2 cm is higher than that of large tumors. We hypothesize that ablation time may influence the ability of RFA to inactivate tumor tissues, because thermal cell death is time-dependent (36, 37). In addition, diffuse echo-blocking of a tumor (fog effect) may occur during ablation, hence affect the success of tumor inactivation (38). Larger tumors may be more susceptible to the fog effect, thus reducing the complete ablation rate.

In this study, the incidence of complications was low. Chen et al. also reported complications after ablation, but only summarized the incidence of skin burns at 4%, and did not report other complications in the study (7). Our study, however, detailed all the complications and their possible cause. Accordingly, skin burn was the most common, with less than 3% incidence rate. This may be due to the small size of the breast, coupled with the proximity of the thin subcutaneous fat layer or tumor tissue to the skin. This also suggests that ablation of tumors smaller than 2cm has fewer complications and is safer than tumors larger than 2cm.Other complications such as skin puckering, skin swelling and chest muscle burn associated with mild heat damage were also observed. Nipple retraction also occurred in some individuals, which may be caused by the proximity of the nipple to the tumor. Of note, one study recorded 40% incidence of complications (26). Here, inflammation of the breast was the main complication (25%), but the inflammations were thought to be “clinically insignificant” by the authors. In this study, breast infections were largely associated with local radiotherapy. In view of treatment related complications, better approaches are undoubtedly needed (39).

Currently, research on RFA in the treatment of breast cancer is in the initial stage, and most experimental designs include surgical resection after ablation, thus findings on RFA alone are very scarce (136 cases). Surgical resection performed after RFA may affect the local recurrence rate because surgical resection after RFA may increase local control rate compared with RFA only. However, in this study, there was no difference in local recurrence rate between the two groups. Only 10 of 17 studies reported whether local recurrence occurred after ablation, and none of the 232 patients developed local recurrence, whether they underwent surgical resection following RFA or not. This may be related to postoperative treatment. Of the 136 patients who only received RFA, 133 patients received postoperative radiotherapy and systemic adjuvant treatment. In addition, most of the patients who only received RFA were hormone receptor positive, HER-2 negative and lymph node negative. Therefore, the lack of local recurrence in patients who did not undergo surgical resection after ablation may not be attributed to the success of RFA alone. Nevertheless, this shows that for small tumors, RFA combined with postoperative radiotherapy and systemic adjuvant treatment may achieve a good local control rate similar to that of surgical operation after RFA. Elsewhere, recurrence was observed in 9 of 564 (6) and 5 of 404 patients that had received RFA (7). The local recurrence of tumors after ablation is related to the rate of tumor cell inactivation after ablation. The complete ablation rate of tumors smaller than 2 cm is higher than that of tumors larger than 2 cm, which also means that the local recurrence rate of tumor less than 2cm is lower when there is no difference in other characteristics of tumor.

This meta-analysis had a few limitations. First, publication bias was found in the studies, possibly because we only included published studies. However, the influence of publication bias was small and the conclusions were reliable as determined by the trim and fill method. Second, the use of minimally invasive treatments is at the foundational stage and the quality of the findings may be weakened by the small sample size (401 cases). Finally, in the analysis of local recurrence rate, RFA alone was performed in 136 cases and the median follow-up time was 29 months. This implies that the conclusion regarding local recurrence rate in early breast cancer following treatment with RFA being 0 should be interpreted with caution. These results illustrate the safety and efficacy of RFA. It can be used for patients unfit for surgery or those unwilling to receive surgical resection of the tumor.

## Conclusion

This meta-analysis shows that the RFA is safe and effective for the treatment of breast cancer with small tumor size with few complications. However, standard adjuvant therapy is needed after operation. Future prospective studies investigating the use of ablation alone in small breast cancer should be conducted to support our findings.

## Data Availability Statement

The original contributions presented in the study are included in the article/**Supplementary Material**. Further inquiries can be directed to the corresponding author.

## Author Contributions

Protocol/project development: L-YX and Q-LH. Data acquisition and interpretation of data: L-YX and W-YX. Statistics analysis of data: L-YX and W-YX. Manuscript drafting: L-YX and W-YX. Manuscript revision and accountable for all aspects of the study: L-YX and Q-LH. All authors contributed to the article and approved the submitted version.

## Conflict of Interest

The authors declare that the research was conducted in the absence of any commercial or financial relationships that could be construed as a potential conflict of interest.

## Supplementary Material

Supplementary Figure 1 Trim and fill plot of publication bias for complete ablation rate **(A)** and complications rate **(B)**.

## Abbreviations

RFA, radiofrequency ablation; H & E, hematoxylineosin; NADH, nicotinamide adenine dinucleotide in its reduced form; CK8, cytokeratine 8.
