# Supplementary figures and images for: Efficacy and Safety of Radiofrequency Ablation for Breast Cancer Smaller Than 2 cm: A Systematic Review and Meta-Analysis
Source: Front Oncol. 2021 May 3;11:651646. doi: 10.3389/fonc.2021.651646 (PMC8126716; doi:10.3389/fonc.2021.651646)

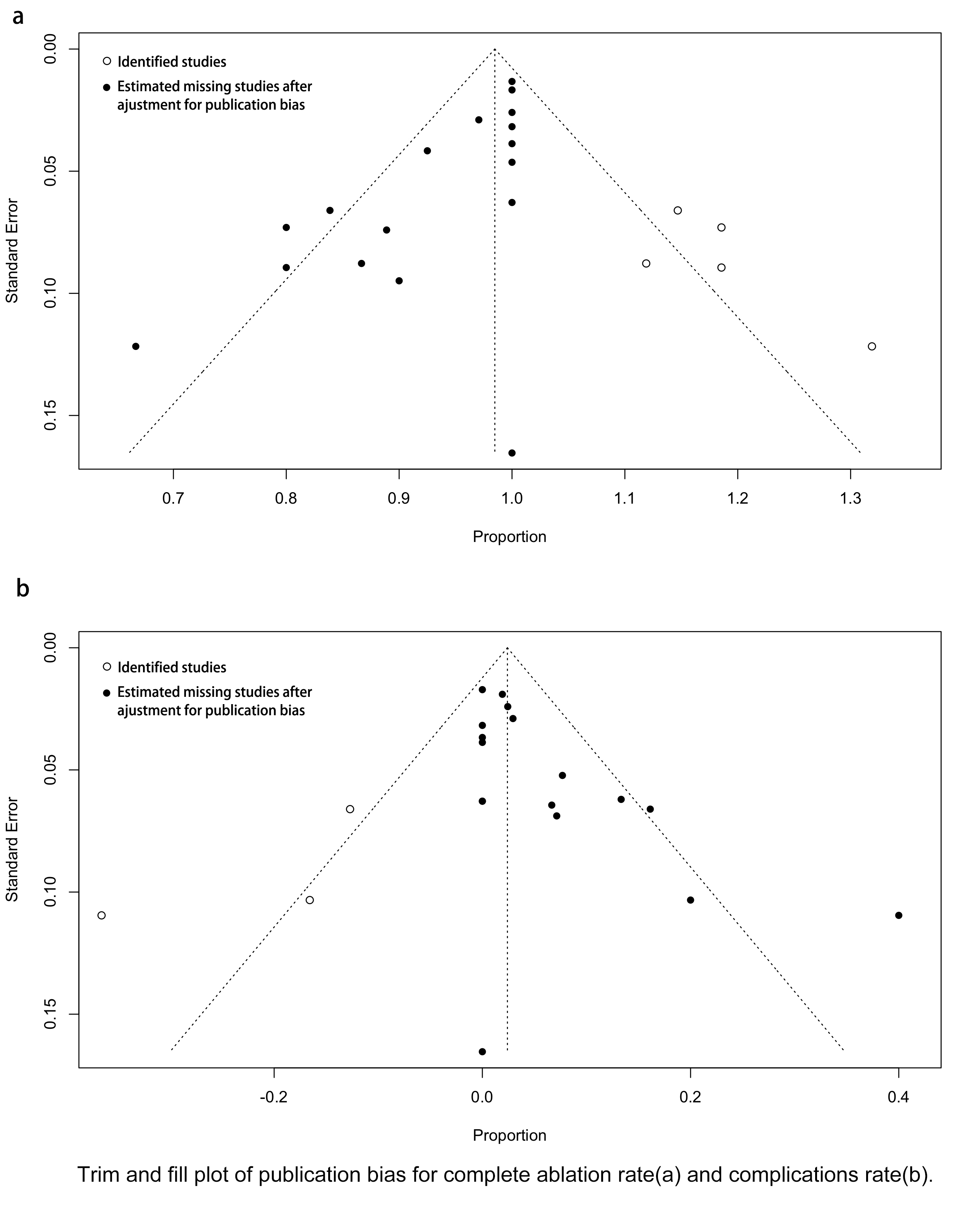

Supplement: Supplementary Figure 1 — Trim and fill plot of publication bias for complete ablation rate (A) and complications rate (B). [file Image_1.jpeg]
