# Supplementary material for: Efficacy and Safety of Radiofrequency Ablation for Breast Cancer Smaller Than 2 cm: A Systematic Review and Meta-Analysis
Source: Front Oncol. 2021 May 3;11:651646. doi: 10.3389/fonc.2021.651646 (PMC8126716; doi:10.3389/fonc.2021.651646)
Supplement: Supplementary file 2 [file Table_1.docx]

Supplementary Table 1. Risk of bias in the included cohort studies (by the MINORS quality assessment tool)

|  | **A clearly stated aim** | **Inclusion of consecutive patients** | **Prospective collection of data** | **Endpoints appropriate to the aim of the study** | **Unbiased assessment of the study endpoint** | **Follow-up period appropriate to the aim of the study** | **Loss to follow up less than 5%** | **Prospective calculation of the study size** | **Total quality scores** |  |
| --- | --- | --- | --- | --- | --- | --- | --- | --- | --- | --- |
| Burak^[10]^ | 2 | 2 | 1 | 2 | 1 | 1 | 2 | 0 | 11 |  |
| Fornage^[11]^ | 2 | 1 | 1 | 2 | 1 | 0 | 2 | 0 | 9 |  |
| Noguch^[15]^ | 2 | 1 | 1 | 2 | 1 | 0 | 2 | 0 | 9 |  |
| Susini^[16]^ | 2 | 1 | 1 | 2 | 1 | 1 | 2 | 0 | 10 |  |
| Khatri^[17]^ | 2 | 2 | 2 | 2 | 2 | 2 | 2 | 0 | 14 |  |
| Oura^[18]^ | 2 | 2 | 1 | 2 | 2 | 1 | 2 | 0 | 12 |  |
| Manenti^[19]^ | 2 | 2 | 1 | 2 | 1 | 1 | 2 | 0 | 11 |  |
| Nagashima^[20]^ | 2 | 2 | 1 | 2 | 1 | 1 | 2 | 0 | 11 |  |
| Wiksell^[21]^ | 2 | 2 | 2 | 2 | 2 | 1 | 2 | 0 | 13 |  |
| Yamamoto^[22]^ | 2 | 2 | 1 | 2 | 1 | 1 | 2 | 0 | 11 |  |
| Ohtani^[23]^ | 2 | 2 | 1 | 2 | 1 | 0 | 2 | 0 | 10 |  |
| Yoshinaga^[24]^ | 2 | 1 | 1 | 2 | 1 | 2 | 2 | 0 | 11 |  |
| Manenti^[25]^ | 2 | 2 | 1 | 2 | 1 | 1 | 2 | 0 | 11 |  |
| Waaijer^[26]^ | 2 | 1 | 1 | 2 | 2 | 1 | 2 | 0 | 11 |  |
| Schässburger^[27]^ | 2 | 1 | 1 | 2 | 1 | 1 | 2 | 0 | 10 |  |
| Nagashima^[28]^ | 2 | 1 | 2 | 2 | 2 | 2 | 2 | 0 | 13 |  |
| García^[29]^ | 2 | 2 | 1 | 2 | 2 | 1 | 2 | 0 | 12 |  |
