# Supplementary material for: Efficacy and Safety of Radiofrequency Ablation for Breast Cancer Smaller Than 2 cm: A Systematic Review and Meta-Analysis
Source: Front Oncol. 2021 May 3;11:651646. doi: 10.3389/fonc.2021.651646 (PMC8126716; doi:10.3389/fonc.2021.651646)
Supplement: Supplementary file 3 [file Table_2.docx]

Supplementary Table 2. Results of Egger’s tests for publication bias

| End point | *t* | *P* |
| --- | --- | --- |
| technically successful ablation rate | -2.25 | 0.04 |
| complete ablation rate | -4.44 | 0.01 |
| complications rate | 3.24 | 0.01 |
